# Supplementation of probiotics in water beneficial growth performance, carcass traits, immune function, and antioxidant capacity in broiler chickens

**DOI:** 10.1515/biol-2021-0031

**Published:** 2021-04-03

**Authors:** Lihuan Zhang, Ruonan Zhang, Hao Jia, Zhiwei Zhu, Huifeng Li, Yueyue Ma

**Affiliations:** College of Life Science, Shanxi Agricultural University, Jinzhong 030801, Shanxi Province, China

**Keywords:** broiler chicks, probiotics, growth performance, carcass traits, gut microbiota

## Abstract

This study aims to investigate the effects of commercial probiotic supplementation in water on the performance parameters, carcass traits, immune function, and antioxidant capacity of broiler chicks. In the experiment, 120 Arbor Acres (AA) broilers (60 male and 60 female) were randomly allocated into four groups (G) – G1: basal diet and G2, G3, and G4: basal diet with 1% *Lactobacillus casei*, 1% *L. acidophilus*, and 1% *Bifidobacterium* in the water, lasting 42 days. The experimental results revealed that probiotic additives produced positive impacts on body weight, average daily feed intake (ADFI), and average daily weight gain for female chicks, whereas these probiotics significantly reduced ADFI and the feed conversion ratio of male chicks (*P* < 0.05). Probiotics efficiently improved eviscerated yield and breast yield while reducing the abdominal fat (*P* < 0.05) for the male broiler chicks. A marked increase was observed in the weight of the spleen, bursa of Fabricius, and thymus in the treatment group (*P* < 0.05). Besides, probiotics produced a significant effect on the concentrations of immune-related proteins (*P* < 0.05) and markedly increased the concentrations of antioxidase and digestive enzymes when compared with the control (*P* < 0.05). The addition of probiotics dramatically reduced the total counts of *Escherichia coli* and *Salmonella* and increased the quantity of *Lactobacilli* (*P* < 0.05). The results of the present study demonstrated an increase in growth performance, carcass traits, immune function, gut microbial population, and antioxidant capacity by supplementing 1% probiotics (*L. casei*, *L. acidophilus*, and *Bifidobacterium*) in the water for broilers.

## 1 Introduction

Chicken meat, which contains unsaturated fatty acids, oleic acid and linoleic acids, reduces low-density lipoproteins and cholesterol, which are harmful to humans. Hence, the large-scale and intensive development of the broiler chicken industry is blooming along with the rising demand for chicken [1]. However, in order to meet the large demand for broilers in the market, antibiotics that promote the growth of broilers are abused [2,3]. Excessive use of antibiotics brings about many problems such as drug resistance in animals and drug residues in livestock products, which threatens the sustainable development of human and nature, and it has emerged as a severe food security issue [4]. To address this problem, several countries have legislated and banned the application of antibiotics as growth promoters in feeds [5]. Consequently, the selection of growth promoters as a replacement for antibiotics has become a hot topic in feed research.

The United Nations Food and Agriculture Organization and the World Health Organization have defined probiotics as “living microorganisms which when administered in adequate amount confer health benefits on the host” [6]. The probiotics, *Lactobacillus*, *Lactococcus*, *Bifidobacterium*, and *Saccharomyces*, are commonly used in laboratory animal experiments [7]. Probiotics are usually produced in the feed industry through the processes of isolation, cultivation, and fermentation and can be used as additives in feed production [8,9]. Multiple works of literature have reported the diverse benefits of probiotic supplementation in breeding commercial animals, including the increase in feed conversion ratio (FCR) and weight gain, egg/milk yield, and the reduction in morbidity and mortality [10,11,12]. Probiotics advance the growth performance, nutrient digestibility, and FCR; improve the gastrointestinal microecological environment; and enhance the internal immunity as well as antioxidant capacity, thereby inhibiting the adhesion of pathogenic bacteria in broiler chickens [13,14,15,16]. In addition, probiotics were used to compact the negative effects of heat stress [8], salmonella [17], detoxification of nitrate [2,16], and aflatoxin [3,18]. The substitute of veterinary drug probiotics therefore is of great significance to enhance the production scale and green development of broilers.

As the previous study reported, multiple probiotics seem to positively affect the production and physiological traits of broiler chickens. However, of which *Lactobacillus casei*, *L. acidophilus*, and *Bifidobacterium* are the most useful probiotics for farmers; and their effect on male and female broilers requires further exploration. To assess the effects of the three probiotics on male and female broilers at different growth stages, the present study aimed to investigate the effects of probiotic additives in the water on growth performance, carcass traits, immune function, gut microbiota, and antioxidant capacity of broilers.

## 2 Materials and methods

### 2.1 Probiotics

In this research, as a probiotic feed additive, *L. casei*, *L. acidophilus*, and *Bifidobacterium* were selected as the experimental strains. The effective live bacteria content of all probiotics was greater than 5 × 10^9^ CFU/g. All bacteria were purchased from Shanghai Xianlong Biotechnology Co., Ltd. (Shanghai, China).

### 2.2 Basal diet and the calculated nutrient composition

The corn–soybean basal diet was prepared according to the National Research Council recommendations (NRC, 1994) and the Chinese Chicken Feeding Standards (2004). Basal diets’ formula and chemical composition are shown in Table 1.

**Table 1:** Composition and nutrient levels of basal diets (on dry matter basis) %

Ingredient (%)	1–21 days of age	22–42 days of age
Corn	55.75	62.00
Soybean meal (44%)	35.00	25.70
Fish meal	3.00	7.00
Soybean oil	3.00	2.50
Salt	0.30	0.20
Limestone	1.00	1.00
CaHPO_4_	1.50	1.00
Choline chloride	0.15	0.10
Mineral premix^a^	0.10	0.20
Vitamin premix^b^	0.20	0.30
Total	100.00	100.00
**Nutrient levels** c		
Metabolizable energy (MJ/kg)	12.47	12.73
Crude protein (%)	21.42	20.46
Calcium (%)	1.01	1.04
Available phosphorus (%)	0.46	0.48
Lysine (%)	1.22	1.18
Methionine (%)	0.35	0.38
Met + Cys	0.71	0.69

a Mineral premix provided per kilogram of diet: Cu (CuSO_4_·5H_2_O) 8 mg, Fe (FeSO_4_·7H_2_O) 50 mg, Zn (ZnSO_4_·7H_2_O) 45 mg, Mn (MnSO4·H_2_O) 65 mg, Se (Na_2_ SeO_3_) 1 mg, Ca(IO_3_)_2_ 1 mg, and Ca_3_(PO_4_)_2_ 0.2 mg.

b Vitamin premix provided per kilogram of diet: VA 40000000 IU, VD_3_ 11000000 IU, VE 80000 IU, VK_3_ 12 g, VB_1_ 10 g, VB_2_ 22 g, VB_6_ 15 g, VB_12_ 100 mg, folic acid 4 g, biotin 300 mg, niacinamide 100 g, pantothenic acid 50 g, and antioxidant 500 mg.

c Nutrition levels were calculated values.

### 2.3 Experimental design

A total of 120 1-day-old Arbor Acres (AA) broiler chicks (60 male and 60 female) were selected for the experiment. On the first day, male and female chicks were identified by the feather sexing method and randomly divided into four groups (30 each) with similar mean body weight (BW), three replicates of each group, and 10 chicks (5 males and 5 females) in each replicate. The broilers were supplied with distilled water with a basal diet as the control group (G1). In the treatment of a single probiotic group, 1% (10 mL of probiotics per liter of distilled water) *L. casei* (G2), *L. acidophilus* (G3), and *bifidobacterium* (G4) were added into daily drinking water. To ensure the activity and the effect of the probiotic preparation, the individual strain was refrigerated at 4°C, and every single strain of probiotic was mixed every morning before use. The whole experimental feeding period was for 42 days.


**Ethical approval:** The research related to animal use has been complied with all the relevant national regulations and institutional policies for the care and use of animals and approved by the Shanxi Agricultural University Ethics Committee.

### 2.4 Feeding management

All the experiments were carried out in a fully enclosed three-tiered chicken coop (length × width × height, 140 cm × 70 cm × 40 cm). The chicken coop was thoroughly cleaned and disinfected prior to the experiment. The environmental conditions in the cage were set following the requirements of the “AA Broiler Breeding Management Manual”. Feeding and drinking water (free from antibiotics) were provided *ad libitum*. The room temperature was kept at 33°C during the first 3 days of age. Thereafter, the temperature was decreased by 3°C per week to reach 24°C at 21 days of age. The temperature was subsequently maintained at ∼24°C until the end of the experiment. Twenty-four hour lighting was provided on day 1, followed by 23 h/day, with 1 h of darkness from 19:00 to 20:00. The chicks were fed regularly, the health status was observed, and the feed consumption of each group was accurately recorded. The chicks were inoculated with Marek’s disease vaccine at the hatchery, infectious bronchitis virus, influenza at day 7, Gumboro disease vaccine at days 14 and 24, and Newcastle disease vaccine at days 7 and 18.

### 2.5 Performance traits

The average BW of each group was measured and recorded at 21 and 42 days of age. The average daily weight gain (ADG), the average daily feed intake (ADFI), and the FCR in each growth period were calculated based on the experimental records. The weight of dead broiler chickens was included to calculate the average weight gain, feed intake, and FCR.

### 2.6 Sample collection and analysis

At 21 and 42 days of age, the broilers were electrically stunned and exsanguinated to obtain tissues. Selected chickens were not given feed 12 h before slaughter, whereas constant access to water was ensured [19]. Five female and five male broilers were randomly selected from each group for slaughtering carcass, characteristic evaluation, artificial anatomy, and weighing. All parameters of live BW, dressed weight, eviscerated weight, half-eviscerated weight (eviscerated weight with giblet), breast muscle, leg muscle, abdominal fat, and immune organ (bursa of Fabricius, thymus, and spleen) were excised and weighed individually. The calculation method of carcass traits was conducted following the description of Ghasemi-Sadabadi et al. [14]. Carcass yield was calculated as a percentage of the pre-live BW of the broilers. The indexes of these immune organs were expressed as immune organ fresh weight (g)/live BW (kg). Five milliliters of arterial blood of the chickens were collected and centrifuged at 3,000× *g* for 15 min to obtain the serum. The jejunum tissue was carefully rinsed with phosphate buffer, and partial tissue was transferred with the serum for preservation at −80°C. For the measurement of the intestinal microbial population, 1 g of the composite gut sample was accurately weighed in a sterile environment.

### 2.7 ELISA and biochemical tests

ELISA kit (Shanghai Meilan, China) was used to determine the concentrations of immune-related proteins (IL-2, IL-10, IgA, and IgG) and digestive enzymes (amylase, lipase, and trypsin) [20]. The contents of superoxide dismutase (SOD), glutathione peroxidase (GSH-Px), total antioxidant capacity (T-AOC), and malondialdehyde (MDA) were determined by biochemical assay on the basis of the instruction of the chemical kits purchased from Nanjing Jiancheng Bioengineering Institute (Nanjing, China). All determination procedures and calculation formulas were carried out in strict accordance with the manufacturer’s instructions.

### 2.8 Gut microbial population

The samples of the chickens were diluted with 9 mL of 0.9% saline solution and mixed in a vortex for 15 min. The viable numbers of bacteria in the samples were subsequently counted by plating serial 10-fold dilutions (in 1% peptone solution) into eosin methylene blue agar, Salmonella–Shigella agar, and lactobacilli de Man, Rogosa, and Sharpe agar plates (to isolate *Escherichia coli*, *Salmonella*, and *Lactobacillus*). *E. coli* and *Salmonella* were aerobically cultured in a 37°C incubator for 24 h before colony counting, and *Lactobacillus* was anaerobically incubated at the same temperature for 48 h after colony counting. Bacterial colony-forming units (CFUs) in the petri dishes were counted using a colony counter. The counts were expressed as log 10 CFUs per gram of digesta (log_10_ CFU/g).

### 2.9 Statistical analyses

The experimental data were compiled by Excel 2016. The data were expressed as mean ± standard error of the mean (mean ± SEM, *n* = 5). The one-way ANOVA using SPSS 20 software (SPSS Inc., IL, USA) was used to analyze the different treatment groups, and Tukey’s test with mean separations was applied to determine the significant differences. A two-tailed *t* test with a *p* value of <0.05 was considered significant.

## 3 Results

### 3.1 Production performance

As shown in Table 2, the supplement of probiotics to daily drinking water had no obvious effect on ADG, ADFI, and FCR of both male and female chickens in their early growth period (0–21 days). From day 22 to 42, the treatment of probiotic efficiently elevated the BW, ADG, and the ADFI in female chickens when compared with the control, whereas no impact was observed on the FCR. In the probiotic group, the ADFI and FCR of male chickens significantly reduced, while BW significantly increased. During the 0–42 days, probiotic supplementation markedly increased the BW, ADG, and ADFI in female chickens and significantly improved BW and FCR in male chickens.

**Table 2:** Effects of probiotic supplementation on the growth performance parameters in male and female broiler chickens

Items	Treatments				Statistical parameters	
	G1	G2	G3	G4	SEM	*p* value
**1–21 days (male)**						
BW, g	507	481	490	517	10.4	0.299
ADFI, g/day	42.7	41.4	41.7	42.0	3.76	0.826
ADG, g/day	21.6	20.1	20.7	22.0	2.35	0.453
FCR, kg/kg	1.98	2.06	2.02	1.91	0.08	0.085
**1–21 days (female)**						
BW, g	569	536	546	578	15.2	0.484
ADFI, g/day	42.65	42.79	42.35	41.59	3.24	0.697
ADG, g/day	24.7	23.2	23.6	25.2	1.56	0.144
FCR, kg/kg	1.73	1.85	1.79	1.65	0.06	0.092
**22–42 days (male)**						
BW, g	1,426^b^	1,527^a^	1,454^b^	1,543^a^	20.1	0.020
ADFI, g/day	125^a^	106^b^	114^b^	109^b^	5.08	0.019
ADG, g/day	43.3	49.8	45.9	48.8	3.91	0.086
FCR, kg/kg	2.60^a^	2.13^b^	2.48^a^	2.23^b^	0.15	0.003
**22–42 days (female)**						
BW, g	1,542^c^	1,787^a^	1,769^ab^	1,824^a^	25.8	<0.0001
ADFI, g/day	115^b^	127^a^	128^a^	128.5^a^	5.26	0.028
ADG, g/day	46.4^b^	59.5^a^	58.2^a^	59.33^a^	4.85^a^	0.037
FCR, kg/kg	2.48	2.14	2.20	2.16	0.18	0.061
**1–42 days (male)**						
BW, g	1,452^b^	1,533^a^	1,462^b^	1,548^a^	21.7	0.031
ADFI, g/day	84.0^a^	73.9^b^	77.8^ab^	75.3^b^	3.12	0.048
ADG, g/day	32.4	34.9	33.2	35.4	2.45	0.069
FCR, kg/kg	2.59	2.12	2.34	2.13	0.11	0.039
**1–42 days (female)**						
BW, g	1,563	1,790	1,764	1,825	23.1	<0.0001
ADFI, g/day	78.9^b^	85.1^a^	85.3^a^	85.0^a^	2.97	0.042
ADG, g/day	35.5^b^	41.4^a^	40.9^a^	42.3^a^	2.86	0.029
FCR, kg/kg	2.19	2.04	2.08	2.01	0.09	0.225

BW: average body weight; ADFI: average daily feed intake; ADG: average daily weight gain; FCR: feed conversion ratio = ADFI/ADG. Mean values (± SEM, *n* = 5) within each row of different treatments with common superscript do not differ significantly at *p* < 0.05. G1: control group, and G2–G4: treatment groups of 1% (probiotic, 10 mL/L water) *L. casei*, *L. acidophilus*, and *Bifidobacterium,* respectively.

### 3.2 Carcass traits

**Figure 1:**
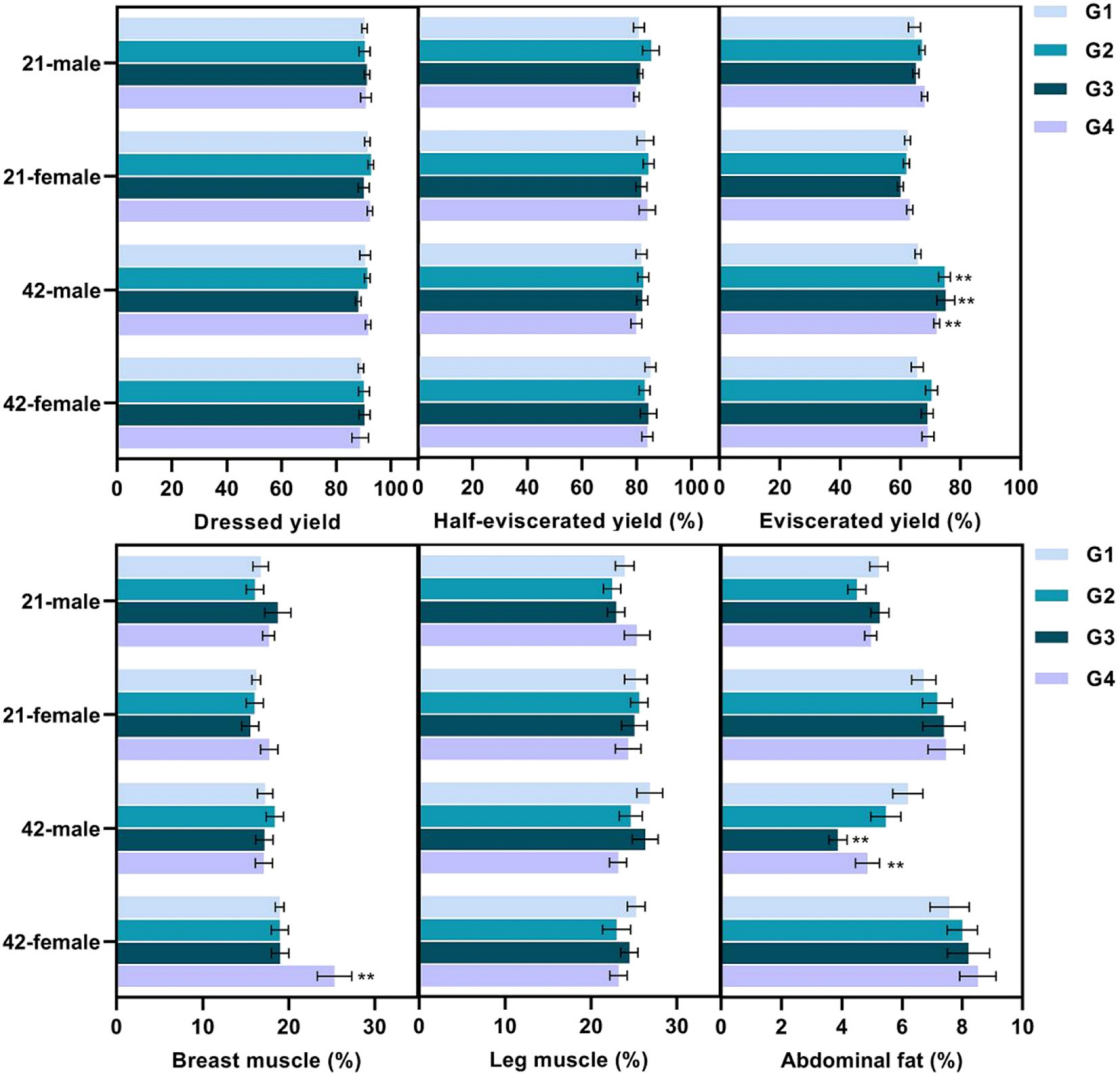
Carcass traits of male and female broilers fed probiotics on days 21 and 42, respectively. These data (mean ± SEM, *n* = 5) were analyzed using one-way ANOVA, followed by *post hoc* Tukey’s test. The “**” indicated when the treatment was compared to the control and *p* < 0.05. G1: control group; G2–G4: treatment groups with 1% (probiotic, 10 mL/L water) *L. casei*, *L. acidophilus*, and *Bifidobacterium*, respectively.

The carcass traits of broilers such as dressed yield, eviscerated yield, half-eviscerated yield, breast muscle, leg muscle, and abdominal fat were tested. As illustrated in Figure 1, no notable difference was revealed amid groups of day 21. On day 42, the probiotic-supplemented male chickens had a significantly higher eviscerated yield, while a dramatically lower abdominal fat percentage than the control. Interestingly, the percentage of chest muscle in female chickens was only significantly increased in the G4 (*Bifidobacterium*) group. No significant difference was observed in half-eviscerated yield and leg muscle. No significant difference was observed in half-eviscerated yield and leg muscle percentage compared with the control group.

### 3.3 Immune function

**Figure 2:**
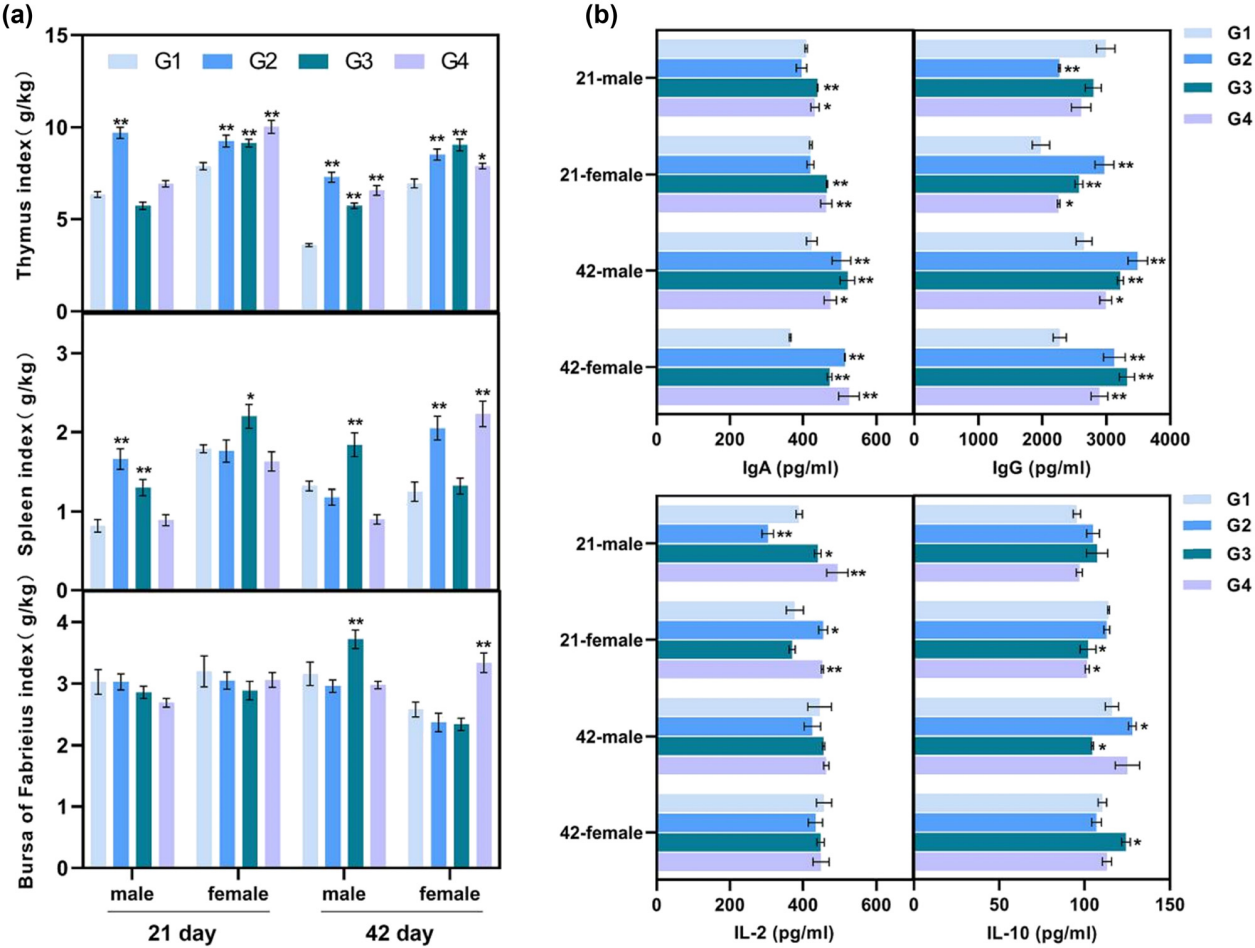
Effects of probiotics on immune function in male and female broiler chickens at 21 and 42 days. (a) The index of lymphoid organs in male and female broiler chickens. Immune organ index = immune organ fresh weight (g)/live weight (kg). (b) The immune-related protein in serum was detected by the ELISA kit. All data (mean ± SEM, *n* = 5) were analyzed using one-way ANOVA followed by *post hoc* Tukey’s test. ** represents the treatment group compared to the control group and indicates *p* < 0.05. * indicates *p* < 0.01. G1: control group and G2–G4: treatment with 1% (probiotic, 10 mL/L water) *L. casei*, *L. acidophilus*, and *Bifidobacterium*, respectively.

The data presented in Figure 2a show that supplemented probiotics produced different effects on the lymphoid organ index of male and female broiler chickens in G2, G3, and G4 groups, respectively. On day 21, compared with the control group, the supplemented probiotics (G2, G3, and G4) markedly increased the thymus index in female broiler chickens, and a dramatic increase was revealed in the spleen index of the male broilers. The G2 manifested a significant increase in the thymus index of male chickens. An increase in the spleen index of the male broilers was observed in the G2 and G3, while that of the female broilers was increased only in the G3. Compared with the control group, the bursa of Fabricius index suggested no significant difference. On day 42, the thymus indexes of male and female broilers in the G2 and G3 groups and the G4 group were significantly higher than those of the control. The spleen index of female broilers in G2 and G4 showed a higher percentage, while male broilers in G3 showed a higher percentage. *L. acidophilus* (G3) had a significant positive impact on the bursa index in male broilers, while, in female broilers, *Bifidobacterium* (G4) had a significant positive impact on bursa index.

Furthermore, the effects of probiotics on concentrations of immune-related proteins (IL-2, IL-10, IgA, and IgG) in broilers’ serum were shown (Figure 2b). On day 21, G3 and G4 had a higher concentration of IgA in male and female broiler chickens; and all three kinds of probiotics significantly enhanced the IgG concentrations in female chickens. *L. acidophilus* (G3) and *Bifidobacterium* (G4) remarkably increased the IL-2 concentration in male chickens, while *L. casei* remarkably decreased the IL-2 concentration. In the female chickens, a significant increase in IL-2 concentration was observed in G2 and G4. The concentration of IL-10 in male chickens in G2 and G3 was higher than that in the control group. However, the concentration of IL-10 in female chickens was decreased in G3 and G4. On day 42, the feeding with probiotics (G2, G3, and G4) greatly enhanced the concentration of IgA and IgG in male and female broilers. The concentration of IL-2 showed no significant difference among all treatment groups. As for roosters, feeding with *L. casei* (G2) significantly increased the IL-10 concentration, while the *L. acidophilus* (G3) significantly decreased the concentration of IL-10 (Figure 2b).

**Figure 3:**
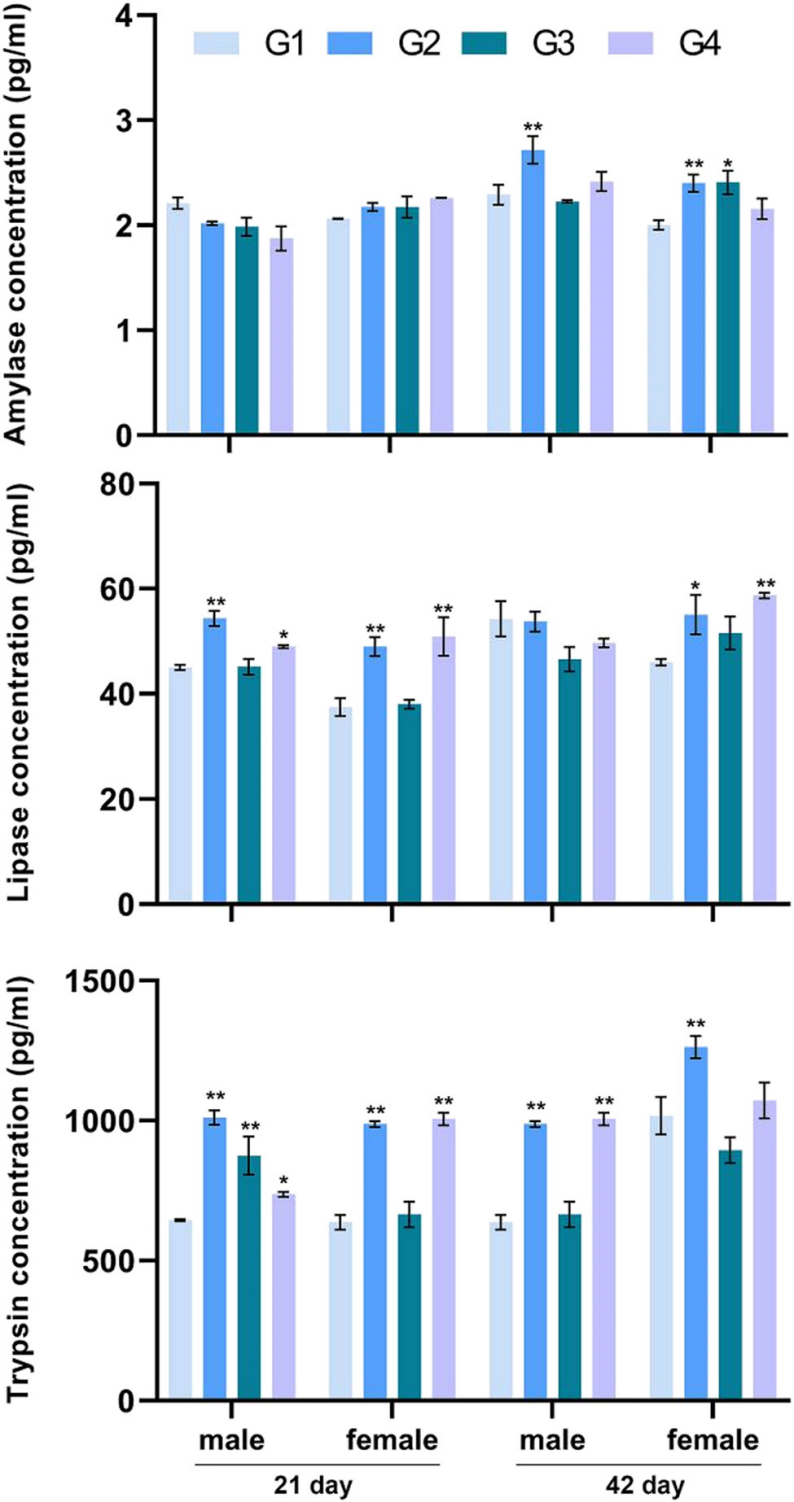
The concentration of digestive enzymes in digestive tract contents of jejunum was detected by the ELISA kit. All data (mean ± SEM, *n* = 5) were analyzed using one-way ANOVA followed by *post hoc* Tukey’s test. ** represents the treatment group compared to the control group and indicates *p* < 0.05. * indicates *p* < 0.01. G1: control group and G2–G4: treatment groups of 1% (probiotic, 10 mL/L water) *L. casei*, *L. acidophilus*, and *Bifidobacterium*, respectively.

### 3.4 Digestive enzymes and gut microbial populations

The lipase contents of the male and female broilers fed with probiotics (G2 and G4) on day 21 were higher than that of the control group (Figure 3). On day 42, the contents of lipase in female chickens fed with *L. casei* (G2) and *Bifidobacterium* (G4) were also significantly increased. In addition, supplementation of *L. casei* (G2) and *Bifidobacterium* (G4) had positive effects on trypsin concentration during the whole age period.

**Figure 4:**
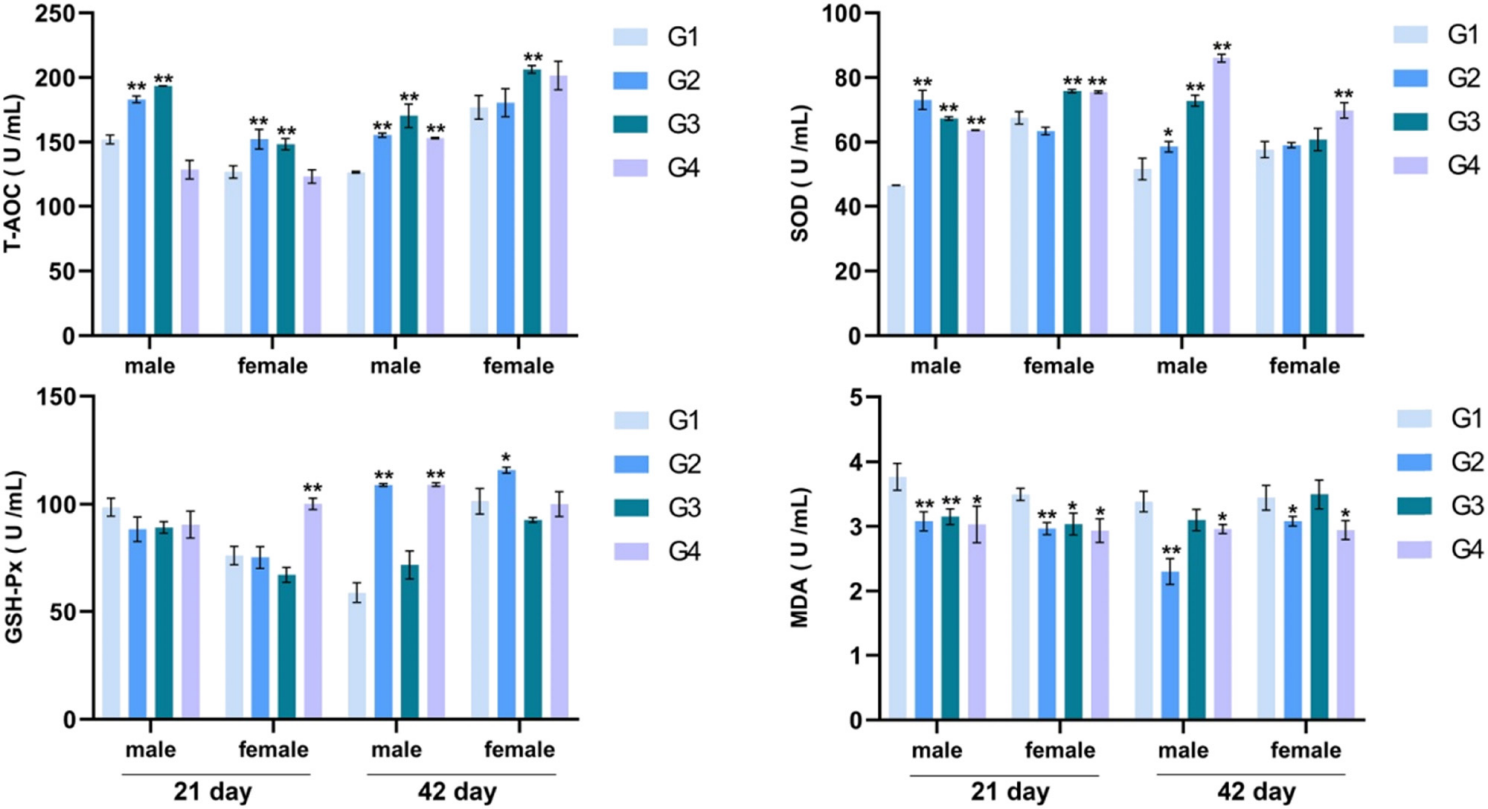
The concentration of antioxidant enzymes and oxidation products in serum was detected by the ELISA kit. All data (mean ± SEM, *n* = 5) were analyzed using one-way ANOVA followed by *post hoc* Tukey’s test. ** represents the treatment group compared to the control group and indicates *p* < 0.05. * indicates *p* < 0.01. G1: control group and G2–G4: treatment groups of 1% (probiotic, 10 mL/L water) *L. casei*, *L. acidophilus*, and *Bifidobacterium*, respectively.

The effects of supplementation of probiotics on gut microbial populations are illustrated in Table 3. On both days 21 and 42, an increase in *Lactobacilli* was observed in the male and female chickens when compared with the control. Of the three probiotic groups, *L. casei* (G2) was the most effective. Additionally, supplementation of probiotics for male and female broiler chickens effectively reduced the number of harmful *E. coli* and *Salmonella* bacteria in the gut.

**Table 3:** Effects of probiotic addition on gut microbial population (log_10_ CFU/g) in male and female broiler chickens at 21 and 42 days

Items	Treatments				Statistical parameters	
	G1	G2	G3	G4	SEM	*p* value
**1–21 day (male)**						
*E*. *coli*	5.02^a^	4.26^c^	4.63^b^	4.30^c^	0.078	<0.0001
*Salmonella*	4.39^a^	4.01^b^	3.98^b^	3.92^b^	0.059	0.0007
*Lactobacillus*	6.46^b^	7.25^a^	6.81^ab^	7.08^a^	0.147	0.0032
**1–21 day (female)**						
*E*. coli	5.25^a^	4.47^b^	5.08^ab^	4.61^b^	0.109	0.0029
*Salmonella*	4.63^a^	4.13^b^	4.22^b^	4.36^b^	0.084	0.0005
*Lactobacillus*	6.04^c^	7.12^a^	6.65^b^	7.03^a^	0.139	<0.0001
**22–42 day (male)**						
*E*. *coli*	5.72^a^	5.04^b^	4.91^b^	5.58^a^	0.136	0.0015
*Salmonella*	4.69^a^	4.48^ab^	4.03^b^	3.92^b^	0.092	0.0024
*Lactobacillus*	6.85^b^	7.49^b^	7.55^a^	6.97^b^	0.164	0.0071
**22–42 day (female)**						
*E*. *coli*	5.36^a^	4.72^b^	5.10^ab^	5.09^ab^	0.141	0.0266
*Salmonella*	4.99^a^	4.41^b^	4.90^a^	3.97^c^	0.121	0.0002
*Lactobacillus*	6.08^c^	7.26^a^	6.54^b^	6.73^b^	0.130	0.0008

Mean values (± SEM, *n* = 5) within each row of different treatments with common superscript do not differ significantly at *p* < 0.05. G1: control group and G2–G4: treatment groups of 1% (10 mL of probiotics per liter of distilled water) *L. casei*, *L. acidophilus,* and *Bifidobacterium,* respectively.

### 3.5 Antioxidative activity

For the analysis of antioxidative activity following broilers being fed with probiotics, the concentration of T-AOC, SOD, GSH-Px, and MDA in serum on 21 and 42 days (Figure 4) was detected as illustrated. The results revealed that the effects of probiotic supplementation on the concentration of SOD and T-AOC were greater than that on GSH-Px. Over half of the treatment groups showed a conspicuous increase in SOD and T-AOC concentration, while changes in GSH-Px concentration were observed in several groups. Moreover, probiotic treatment alone significantly reduced the concentration of MDA in the male and female broilers in contrast with the control at 21 days of age. Feeding with *L. casei* (G2) or *Bifidobacterium* (G4) intensely reduced MDA concentration on day 42. These data demonstrated that probiotic supplementation positively affected antioxidant activity in broilers.

## 4 Discussion

The multistrain probiotic containing 1 × 10^8^ CFU/g of *L. casei*, *L. acidophilus*, *B. thermophilum*, and *Enterococcus* fed (0.900 g/kg) to broilers had positive effect on chickens [21]. The *L. casei* P-8 was added to the chickens’ drinking water at a final concentration of 2 × 10^6 ^CFU/mL and showed improved weight gain, feed intake, and feed efficiency [20]. The probiotic mixture (*L. acidophilus*, *L. casei*, *Bifidobacterium bifidum*, and *Enterococcus faecium*) supplement at 2 × 10^8^ CFU/kg improved FCR [22]. In summary, probiotics are beneficial to broiler growth when the density of the viable bacteria is greater than 10^5^ CFU/g. In this study, the density of viable bacteria of *L. casei*, *L. acidophilus*, and *Bifidobacterium* was greater than 5 × 10^9^ CFU/mL. One percent probiotic supplement in water could ensure that probiotics have effect on broiler growth. These findings confirmed the positive effects of probiotics on the growth performance of broilers, consistent with the previously reported studies [15,23,24]. Furthermore, the supplement of single probiotic or mixed probiotic feed additives could remarkably improve broilers’ growth performance and reduce their FCR under normal, stress, disease, and other challenging conditions [2,3,8,16,17,18]. Broilers fed with the *L. casei* and *L. acidophilus* exhibited an increase in ADG [25]. Conversely, others have stated that probiotics had no positive effect on broilers’ performance [26]. Our data indicated that the addition of probiotics in water significantly improved the FCR of male broilers, while no significant effect was observed on FCR in female broilers. The inconsistent roles of probiotics among these studies including the present study may be related to the type, dosage of probiotics, and the breed of the broilers. The positive effects of *L. casei* and *Bifidobacterium* on the growth performance of broilers were higher than that of *L. acidophilus* in our study. It is pertinent to mention that FCR is very crucial in the poultry sector as it reflects the efficient utilization of nutrients [27]. The amylase, protease, and lipase produced by probiotic preparation could degrade plant carbohydrates and complex substances in the feed, so that the carbohydrates were better absorbed and utilized by the intestine, thereby improving the feed efficiency [28]. In the present study, the treatment of probiotics increased the concentration of digestive enzymes in the jejunum of broilers in varying degrees, indirectly interpreting possible reasons for FCR improvement. Low FCR treated by *L. casei* and *Bifidobacterium* seemed to be related to a high concentration of digestive enzymes.

As a probiotic, *lactobacillus* is suitable for domestic animals, because it can inhibit the growth of pathogenic bacteria and promote the growth of nonpathogenic bacteria by producing different metabolites, thereby improving the intestinal microecological environment [2,3,8,16,17,18]. The commonly encountered pathogenic or zoonotic bacteria in poultry farming are *E. coli*, *Salmonella enterica*, *Campylobacter jejuni*, and *Clostridium perfringens* [5]. In the present study, supplementation of *L. casei*, *L. acidophilus*, and *Bifidobacterium* in water reduced the abundance of *E. coli* and *Salmonella* and increased the abundance of *Lactobacillus*. The results were consistent with the findings of other researchers who had observed an improvement in the intestinal microbial population in broilers fed with probiotics [29,30]. In general, supplementation of probiotics in water could ameliorate the structure of the gut microflora.

Eviscerated yield, breast muscle yield, leg muscle yield, and abdominal fat rate were essential indicators for evaluating the slaughtering performance of broilers. The addition of probiotics increased the carcass yield of broiler chickens as previously reported [14,15]. Ghasemi-Sadabadi et al. (2019) reported that the supplement of probiotic mix (*L. acidophilus*, *L. casei*, and *B. thermophilum*) had a significant effect on the carcass yield, thigh yield, and abdominal fat in male and female Ross 308 chickens [14]. On the contrary, Qorbanpour et al. (2018) reported that the weights of carcass, breast, and thighs in chickens were not significantly influenced by dietary treatments with multistrain probiotics (*L. acidophilus*, *L. casei*, *E. faecium*, and *B. thermophilum*) [21]. Therefore, we chose to add a single *L. acidophilus*, *L. casei*, and *B. thermophilum* to explore the influence of probiotic on broiler chickens. Our findings revealed that the addition of probiotics effectively increased the eviscerated yield and reduced the abdominal fat rate in male broilers. Hence, it seems that the use of probiotic increased digestion and absorption due to higher intestinal microbial population and gut health, and the balance of absorbed nutrients increased and caused decreasing abdominal fat. However, no significant difference was observed in dressed yield, breast yield, and thigh yield. The difference in carcass traits between male and female broilers was probably related to the utilization of nutrients under different probiotic treatments. Differences in nutrient utilization between male and female broilers were previously reported [31]

The lymphatic organs of poultry are the spleen, bursa of Fabricius, and thymus, and their weight directly reflects the strength of the internal immune function [32]. It has been reported that single probiotic treatment or in combination can increase the weight of the spleen, thymus, and bursa of Fabricius in broilers [33]. In the present study, different probiotics exerted the greatest impact on the thymus index and spleen index of broilers. Lactic acid bacteria were widely reported to enhance the immune system of animals by positively affecting the innate and adaptive immune responses [34]. The contents of IgG and IgA in serum are important markers to estimate the changes in animal immune function. The administration of *Lactobacillus* spp. could efficiently activate the immunity of mucosa in chickens by increasing the levels of IgA and IgG [35]. These confirmed our results that the treatment of probiotics increased the levels of IgG and IgA in the serum of broilers at all growth stages. IL-2 and IL-10 were the cytokines that reflected the immune level secreted by Th1 and Th2 cells [36]. Wang et al. (2015) reported that *L. plantarum* strain P-8 activated the protective immune response in broilers, and the upregulated IL-2 and the downregulated IL-10 transcriptions were detected in an age-dependent manner [20]. Another study reported that feeding broilers with *Bacillus subtilis* increased the expression of IL-2 and IL-10 [37]. In this study, IL-2 concentration increased while the IL-10 concentration increased or decreased in different treatment groups. These results suggested that feeding with probiotics probably had an effect on broiler chickens’ immune function. Besides, the different effects of probiotics on the immune system of broilers were probably correlated with the differences in fermentation substrates and products of the three probiotics, resulting in different degrees of immune response between male and female broilers.

To eliminate excessive free radicals, levels of antioxidant enzymes scavenging free radicals *in vivo* will increase and indirectly reflect the degree of oxidative stress [38]. SOD, CAT, MDA, and GSH-Px are all antioxidant enzymes. The ability of the human body to scavenge free radicals can be determined by measuring its activity [38]. T-AOC level reflects the total antioxidant level of various antioxidants and antioxidant enzymes in the measurement. MDA is a product of lipid peroxidation, and its content reflects the degree of lipid peroxidation [39]. Therefore, the content and activity of the antioxidant enzymes and lipid peroxidation products in serum can be determined by comprehensively evaluating the capacity of additive antioxidants [40]. Several studies have reported that lactic acid bacteria can affect the activity and content of antioxidant enzymes in the body and reduce oxidative stress damage in the intestine [13,41,42]. We had observed significant increases in SOD and T-AOC concentration and a significant decrease in MDA concentration. These results indicated that probiotics had an obvious antioxidant effect on broilers, which was consistent with the results of relevant studies [2,3,8,16], but its mechanism needs further studying.

## 5 Conclusions

In summary, broilers offered with *L. casei*, *L. acidophilus*, and *Bifidobacterium* at 1% diets containing greater than 5 × 10^9^ CFU/g greatly improved their growth performance, carcass traits, their immune function, and antioxidant capacity to a great extent. *L. casei* and *Bifidobacterium* had a higher positive effect on the growth performance of male and female broilers compared to *L. acidophilus*.
